# Altered distribution and localization of organellar Na^+^/H^+^ exchangers in postmortem schizophrenia dorsolateral prefrontal cortex

**DOI:** 10.1038/s41398-023-02336-2

**Published:** 2023-02-02

**Authors:** Brandon S. Pruett, Anita L. Pinner, Pitna Kim, James H. Meador-Woodruff

**Affiliations:** University of Alabama at Birmingham, Birmingham, AL USA

**Keywords:** Molecular neuroscience, Schizophrenia

## Abstract

Schizophrenia is a complex and multifactorial disorder associated with altered neurotransmission as well as numerous signaling pathway and protein trafficking disruptions. The pH of intracellular organelles involved in protein trafficking is tightly regulated and impacts their functioning. The SLC9A family of Na^+^/H^+^ exchangers (NHEs) plays a fundamental role in cellular and intracellular pH homeostasis. Four organellar NHE isoforms (NHE6-NHE9) are targeted to intracellular organelles involved in protein trafficking. Increased interactions between organellar NHEs and receptor of activated protein C kinase 1 (RACK1) can lead to redistribution of NHEs to the plasma membrane and hyperacidification of target organelles. Given their role in organelle pH regulation, altered expression and/or localization of organellar NHEs could be an underlying cellular mechanism contributing to abnormal intracellular trafficking and disrupted neurotransmitter systems in schizophrenia. We thus characterized organellar NHE expression, co-immunoprecipitation with RACK1, and Triton X-114 (TX-114) phase partitioning in dorsolateral prefrontal cortex of 25 schizophrenia and 25 comparison subjects by Western blot analysis. In schizophrenia after controlling for subject age at time of death, postmortem interval, tissue pH, and sex, there was significantly decreased total expression of NHE8, decreased co-immunoprecipitation of NHE8 (64%) and NHE9 (56%) with RACK1, and increased TX-114 detergent phase partitioning of NHE6 (283%), NHE9 (75%), and RACK1 (367%). Importantly, none of these dependent measures was significantly impacted when comparing those in the schizophrenia group on antipsychotics to those off of antipsychotics for at least 6 weeks at their time of death and none of these same proteins were affected in rats chronically treated with haloperidol. In summary, we characterized organellar NHE expression and distribution in schizophrenia DLPFC and identified abnormalities that could represent a novel mechanism contributing to disruptions in protein trafficking and neurotransmission in schizophrenia.

## Introduction

Schizophrenia (SZ) is associated with significant economic burden and early mortality, despite available antipsychotic medications which do not adequately treat negative symptoms or cognitive decline [1, 2]. Thus, a better understanding of the molecular pathophysiology of SZ is needed to target future therapies. Widespread alterations in protein post-translational modification (PTM) and trafficking are consistently reported in SZ brain including disruptions in endoplasmic reticulum (ER) protein processing [3, 4], trafficking of AMPA receptor subunits [5–7], and altered protein degradative pathways [8, 9]. Further, glycosylation of glutamate and GABA receptor subunits and transporters is altered in SZ brain [10–12] with abnormal glycosylation of GABA_A_ receptor subunits being linked to disruption of their trafficking [3]. Despite alterations in protein PTM and trafficking in SZ brain, no common feature underlying these deficits has been clearly identified. Of note, the pH within organelles most directly involved with protein modification, intracellular trafficking, and the secretory pathway such as ER, Golgi, and endosomes is very tightly regulated, and disruptions to the pH of these compartments can greatly impact their function [13]. For instance, lowered intracellular pH causes reversible disassembly of Golgi and disrupts trafficking between ER and Golgi [14]. In addition, disruptions in Golgi pH lead to altered glycosylation and membrane trafficking of proteins [15], while many cellular processes involved in endocytic trafficking and recycling at the synapse are also sensitive to pH alterations [16]. Thus, changes in organellar pH regulation could contribute to protein PTM and trafficking abnormalities in SZ brain.

**Fig. 1:**
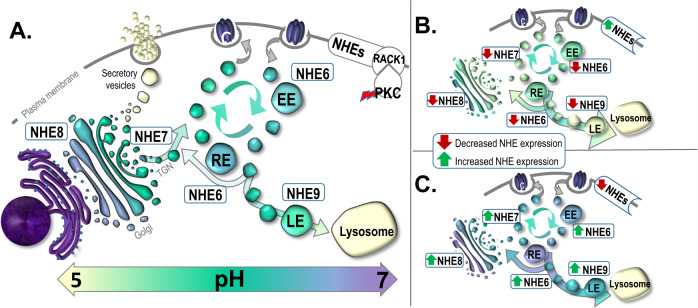
Schematic representation of organellar Na^+^/H^+^ exchanger (NHE) distributions and roles in intracellular compartmental pH regulation. **A** Organellar NHEs play an integral role in regulating the pH of the organelles involved in protein post-translational modification (PTM) and trafficking. NHE6 and NHE9 are localized to early/recycling and late endosomes, respectively while NHE7 and NHE8 are localized to trans-Golgi network and mid/trans-Golgi stacks, respectively. There is an interplay between NHEs at organelle targets and the plasma membrane (PM) with NHEs being stabilized at the PM by interactions with receptor of activated protein C kinase 1 (RACK1), which is activated by protein kinase C (PKC). Altered expression or distribution of organellar NHEs impacts organelle pH regulation and disrupts protein PTM and trafficking with (**B**) decreased expression or activity at organelle targets typically leading to acidification, and (**C**) increased expression or activity at organelle targets typically leading to alkalinization. EE early endosome, LE late endosome, RE recycling endosome, TGN trans-Golgi network.

The family of Na^+^/H^+^ exchangers (NHEs) is a major regulator of intracellular and organellar pH. Each of the nine known NHEs is targeted to a specific intracellular location, where it functions to reduce luminal acidity by exchanging hydrogen for sodium and/or potassium ions [17, 18]. NHE1-5 are targeted to the plasma membrane (PM), while NHE6-9 are targeted to intracellular organelles. NHE6 and NHE9 are primarily localized to early/recycling and late endosomes, respectively, while NHE7 and NHE8 are localized to the trans-Golgi network (TGN) and mid/trans-Golgi stacks, respectively [19–22] (Fig. 1). The NHEs have been implicated in myriad neurodevelopmental and neuropsychiatric disorders. Of the PM NHEs, NHE1 is predominantly expressed in brain, and NHE1 mutations are associated with epilepsy, ataxia, and growth retardation [18]. Of the organellar NHEs, NHE6, NHE7, and NHE9 have all been linked to neurodevelopmental illness. Loss of function mutations in X-linked NHE6 cause Christianson syndrome (CS) in affected males, characterized by postnatal microcephaly, nonverbal status, moderate to severe intellectual disability, epilepsy, ataxia, hyperkinesis, and symptoms of autism [23–25]. A likely gain-of-function missense mutation in NHE7 is associated with alkalinization of Golgi and altered protein glycosylation, and it has been implicated in nonsyndromic X-linked intellectual disability in affected males [26]. Disruptions in NHE9 have been associated with autism, epilepsy, addiction, and attention-deficit/hyperactivity disorder [18]. In addition, idiopathic autism is associated with NHE6 and NHE9 gene expression changes [27]. Thus, NHEs, particularly organellar NHEs, are associated with neurodevelopmental disorders. Given the significant genetic overlap between autism and schizophrenia [28, 29], exploring the potential role of organellar NHEs in SZ is warranted.

There is evidence linking altered organellar NHE regulation to SZ, including decreased gene expression of NHE6 and NHE7 in induced pluripotent stem cell (iPSC)-differentiated neurons derived from individuals with SZ [30]. In addition, female carriers of NHE6 mutations associated with CS have an attenuated but varied phenotype including an 8-25 fold higher prevalence of primary psychotic disorders than the general population, with at least one report of a female carrier with childhood-onset SZ [25, 31, 32]. One study identified a missense variant of NHE6 in a female SZ patient as a rare X-linked variant associated with this illness [28]. Although organellar NHEs have not been identified in SZ genome-wide association studies (GWAS), there is evidence that regulation of organellar NHEs is impacted by genes that have been directly identified in SZ GWAS. For instance, knockdown of one such gene, neurogranin [33–35], is associated with changes in NHE9 protein expression and NHE6 phosphorylation [36]. Despite this evidence suggestive of altered organellar NHEs in SZ, these transporters have not been systematically characterized in SZ brain.

Organellar NHEs can directly impact intracellular and organellar pH regulation through either altered expression or intracellular distribution (Fig. 1B/C). For example, increased interaction of NHE6 with the cytoplasmic scaffolding protein receptor of activated protein C kinase 1 (RACK1), a target of protein kinase C (PKC), leads to redistribution of NHE6 from endosomes to the PM and endosomal over-acidification [37, 38] (Fig. 1B). In cancer cells, redistribution of NHE6 to the PM is triggered by hypoxia [38], which causes a change in energy metabolism termed the Warburg effect [39]. This is a shift from the Krebs cycle and oxidative phosphorylation toward increased glycolysis, leading to accumulation of lactate and increased acidity [39]. Thus, redistribution of NHE6 to the PM under these conditions may serve as a compensatory mechanism for managing reduced intracellular pH but at the expense of organellar pH regulation and function.

Similar alterations in energy metabolism are also seen in SZ brain [40]. There is a well-documented increase in lactate and decrease of pH across multiple brain regions in SZ postmortem tissue [40–46], which is also seen in drug-naïve genetic animal models with relevance to SZ [45] suggesting that these findings are not functions of antipsychotic treatment or postmortem artifacts. In vivo magnetic resonance spectroscopy (MRS) studies also demonstrate comparable findings. Lactate as measured by MRS is increased in anterior cingulate cortex of SZ patients [47] with this finding being more pronounced with increasing illness duration [48] and negatively correlated with general cognitive function and functional capacity [47]. In addition, MRS-measured intracellular pH is reported across several studies to be decreased in the frontal lobes of SZ patients [49–51], and this has been correlated with emotional withdrawal in SZ [52]. Thus, there is significant overlap between energy metabolism changes in SZ brain and the Warburg effect in cancer biology.

Given that energy metabolism changes similar to those in SZ brain have been associated with redistribution of NHE6 to the PM, that organellar NHEs are associated with neurodevelopmental illness including increased risk of psychotic illness, and that organellar NHEs play a vital role in the pH regulation of organelles in the secretory pathway known to be dysregulated in SZ brain, we sought in this study to characterize the expression and intracellular distribution of organellar NHEs as well as their interactions with RACK1 in SZ postmortem dorsolateral prefrontal cortex (DLPFC).

## Methods

### Tissue acquisition and preparation

Full thickness gray matter from postmortem DLPFC (Brodmann areas 9/46) of 25 patients diagnosed with SZ and 25 age and sex-matched comparison (COMP) subjects was acquired through the NIH NeuroBioBank from the Icahn School of Medicine at Mount Sinai Brain Collection in compliance with the Icahn School of Medicine at Mount Sinai Institutional Review Board protocol, and with next of kin consent obtained for each subject. Tissue from DLPFC was chosen as it is implicated in cognitive impairment in SZ [53–56] and numerous protein PTM and trafficking deficits have been reported in this region in SZ [4, 5, 10–12, 57, 58]. Subjects from this collection were recruited prospectively and underwent extensive antemortem diagnostic and clinical assessments. SZ patients were diagnosed using DSM-III-R criteria with two experienced clinicians agreeing upon this diagnosis. In addititon, each subject had a documented history of psychotic symptoms prior to age 40 and at least 10 years of psychiatric hospitalization with a diagnosis of SZ. Exclusionary criteria included a history of substance abuse, death by suicide, coma for more than 6 h prior to death, or neuropathological evidence of neurodegenerative disease. Human frontal cortex provided by the Alabama Brain Collection was also utilized for optimization of protocols and validation of antibodies prior to use in SZ and COMP study subject tissue. Brain tissue was dissected, snap frozen in liquid nitrogen, and stored at −80 °C prior to homogenization and use. SZ and COMP groups (*n* = 25 per group) were well-matched for age at time of death (mean ± SD, COMP: 70.7 ± 15.7 years, SZ: 68.9 ± 11.3 years), postmortem interval (PMI; mean ± SD, COMP: 13.0 ± 6.7 h, SZ: 14.2 ± 5.3 h), tissue pH (mean ± SD, COMP: 6.5 ± 0.3, SZ: 6.4 ± 0.2), and sex (M:F, COMP: 18:7, SZ: 20:5) as detailed in Supplementary Table 1.

### Antipsychotic-treated rats

Animal studies and procedures were performed in accordance with institutional guidelines and approved by the Institutional Animal Care and Use Committee of the University of Alabama at Birmingham. Either haloperidol decanoate (28.5 mg/kg) or vehicle (sesame oil) was administered over 9 months to male Sprague-Dawley rats (250 g) housed in pairs (*n* = 10 per group). Treatment was every 3 weeks by intramuscular injection totaling 12 injections [59, 60]. Brains were harvested following rapid decapitation. Dissections of the right frontal cortex were done on wet ice, snap frozen, and stored at −80 °C. Samples were randomized and experimenters were blinded until data analyses. Sample sizes were chosen based on variability of similar measures in previous studies to yield sufficient power to detect a 20% difference between groups [61].

### Tissue homogenization

Tissue samples were homogenized in cold 5 mM Tris-HCl pH 7.5, 0.32 M sucrose with a protease inhibitor tablet and a phosphatase inhibitor tablet (Complete Mini, EDTA-free and PhosSTOP both from Roche Diagnostics, Mannheim Germany). A Power Gen 125 (Thermo Fisher Scientific, Rockford, Illinois) homogenizer was used at speed setting #5 for 60 s. Protein concentration was determined using a BCA protein assay kit (Thermo Scientific, Rockford, Illinois). After homogenization, samples were stored at −80 °C until used for assay.

### Western blot analysis

Samples were denatured in sample buffer (28.3 mM Tris-hydrochloride pH 6.8, with 6% glycerol, 0.75% sodium dodecyl sulfate (SDS), and 0.4% beta-mercaptoethanol (BME)) at 70 °C for 10 min then stored at −20 °C. Samples were loaded onto NuPAGE 4–12% Bis-Tris gels (Invitrogen, Carlsbad, CA) and transferred to nitrocellulose membranes using a BioRad Semi-Dry Transblotter (Hercules, CA). Membranes were incubated using the conditions indicated in Supplementary Table 2. For a blocking experiment to determine the specificity of NHE6 antibody immunoreactive bands, incubation with NHE6 antisera (Abcam, ab137185) was done in the presence and absence of 5× the amount of NHE6 recombinant protein (Abcam, ab161011). After incubation in primary antisera, membranes were washed in cold Tris-buffered saline + 0.05% Tween-20 (TBST) before being probed with infrared dye-labeled secondary antibody diluted with 50% LI-COR/50% TBST blocking buffer for 1 h at room temperature. Finally, membranes were washed in cold TBST, then briefly rinsed in MilliQ water before being scanned with a LI-COR Odyssey imager. All antibodies/antisera were optimized for ideal conditions for each target protein within the linear range of detection for each assay and ensuring the primary antibody was present in excess (Supplementary Table 2). Valosin-containing protein (VCP) was unchanged in multiple regions of schizophrenia brain [62, 63] and was used as an intralane loading control for Western blot normalization.

### RACK1 co-immunoprecipitation

RACK1 antibody (BD Biosciences, 610178, mouse monoclonal) or pre-immune, non-specific mouse IgG (Vector Laboratories, I-2000) was bound to TBST-washed Invitrogen M-280 sheep anti-mouse IgG Dynabeads (Thermo Fisher Scientific, 11202D) for 30 min at 4 °C on a rotisserie, and unbound antibody was removed by rinsing with TBST. Homogenates were then incubated with the TBST-washed, antibody-bound beads for 1 h at 4 °C on a rotisserie, and unbound lysate (supernatant) was removed and beads washed with TBST. Bead-bound proteins were eluted with 2× reducing sample buffer (57 mM Tris-hydrochloride with 12% glycerol, 1.5% SDS, and 0.8% BME brought to a pH of 6.8) at 70 °C for 10 min.

### Triton X-114 phase separation

Diluted solutions of the nonionic detergent Triton X-114 (TX-114) will separate into aqueous and detergent phases when subjected to temperatures above 20 °C [64], with hydrophilic proteins partitioning into the aqueous phase and amphiphilic proteins partitioning into the detergent phase. Samples were diluted to a total protein concentration of 0.2 μg/μL in homogenization buffer. 400 μL (80 μg of total protein) of each sample was briefly vortexed with 100 μL TX-114 and then incubated at 37 °C for 1 h until a clear interface was visible. The top aqueous layer was transferred to a separate tube, and ice-cold phosphate-buffered saline (PBS) was added to both phases to bring the total sample volume of each phase to 800 μL. Next, 700 μL of chilled acetone (−20 °C) was added to each sample, and they were stored overnight at −20 °C. Samples were then pelleted at 15,000 × *g* at 4 °C for 30 min and re-suspended in 50 μL of PBS containing protease inhibitor tablets (Roche). Protein concentrations were determined by BCA assays (Thermo Fisher). Samples were stored at −20 °C until processed for Western blot analyses.

### Data analysis

Protein expression was determined using LI-COR Image Studio Lite Version 5.2 (Lincoln, NE). Intensity values were normalized to intralane VCP intensity values for total expression, normalized to intralane RACK1 expression for co-immunoprecipitations (co-IPs), or normalized to PSD95 or GAPDH for detergent and aqueous TX-114 phase partitioning, respectively. Total VCP was not changed between SZ and COMP groups (data not shown), consistent with previous reports [62]. For all dependent measures, outliers were detected by the ROUT method [65] and removed.

Data were analyzed using a univariate general linear model (GLM) approach in order to control for potentially confounding factors (age, PMI, tissue pH, and sex). Preliminary GLMs including subject age, PMI, and tissue pH as covariates and sex as a fixed factor but not including group (COMP and SZ) were run to determine which potentially confounding factors were associated with our dependent measures. Any factor found in the preliminary GLMs to be associated with our dependent measures with a *p* < 0.1 was then included in a subsequent GLM along with group and the interaction between each confounding factor and group. With the exception of group, covariates, fixed factors, and interaction terms with *p* ≥ 0.05 were then sequentially removed from the GLMs starting with the least significant factor until the only factors retained in the GLMs were significant at *p* < 0.05. The confounding factors included in the final GLMs for the main analysis can be found in Supplementary Table 3. These GLMs were then used to assess for group level differences in our dependent measures while controlling for significantly confounding factors. Estimated marginal means derived from these GLMs were calculated with covariates set to their median values (Table 1). The effect size measure partial Eta squared (η^2^_*p*_) was also calculated from the GLMs with η^2^_*p*_ = 0.01, η^2^_*p*_ = 0.06, and η^2^_*p*_ = 0.14 generally accepted as the levels for small, medium, and large effect sizes, respectively [66]. While GLMs are fairly robust even when assumptions of normality and homogeneity of variance are violated, we realized that in some instances the ratio of variances was above the standard rules of thumb for robustness (variance ratio ≤ 3) (Table 1). For this reason, we replicated the normal theory-based analyses with distribution-free analyses using estimates based on the bootstrap. The bootstrap is an assumption-free resampling approach based on the empirical distribution of the test statistics that yields accurate test statistics and *p* values even when normal theory assumptions are violated (e.g., when error terms are not normally distributed and/or within-group variance differs between groups) [67–69]. All bootstrap statistics are based on 1000 bootstrap samples.

**Table 1:** NHEs and RACK1 in schizophrenia and comparison dorsolateral prefrontal cortex: summary of results.

Assayed protein			Raw Data							GLM								
			COMP			SZ			Ratio of Variance	COMP		SZ		F	*p*	Effect Size	Bootstrap	DOC
			Mean	SEM	N	Mean	SEM	N	(SZ:COMP)	EMM	SEM	EMM	SEM			η^2^_*p*_	*p*	
Total expression	NHE6	GO	2.4	0.2	24	2.5	0.2	24	1.2	2.4	0.2	2.5	0.2	0.20	0.66		0.64	
		HGM	0.41	0.04	25	0.38	0.04	25	0.64	0.41	0.04	0.38	0.04	0.43	0.52		0.54	
		CGM	1.31	0.07	25	1.40	0.08	25	1.1	1.30	0.07	1.42	0.07	1.4	0.25		0.27	
	NHE7		1.9	0.2	25	1.5	0.1	25	0.68	1.8	0.1	1.6	0.1	1.9	0.18		0.19	
	NHE8		23.4	4.3	24	9.2	1.7	20	0.12	21.8	3.0	9.2	3.3	4.1	0.05	0.09	**0.02**	**↓**
	NHE9		1.2	0.1	24	1.2	0.1	25	0.72	1.2	0.1	1.2	0.1	0.21	0.65		0.65	
	RACK1		1.1	0.1	25	1.0	0.1	25	1.1	1.0	0.1	1.0	0.1	0.08	0.78		0.77	
RACK1 Co-IP	NHE6		0.088	0.008	25	0.087	0.008	25	0.77	0.088	0.008	0.087	0.008	0.02	0.90		0.91	
	NHE7		0.35	0.06	21	0.20	0.03	22	0.24	0.33	0.04	0.20	0.04	3.5	0.07	0.09	0.06	↓
	NHE8		12.4	2.5	22	5.3	1.0	23	0.18	10.2	1.4	3.7	1.5	**13.4**	**0.001**	**0.25**	**0.002**	**↓**
	NHE9		0.009	0.001	25	0.004	0.001	25	0.15	0.009	0.001	0.004	0.001	**13.6**	**0.001**	**0.22**	**0.002**	**↓**
TX-114 phase separation	NHE6	Aqueous	0.010	0.002	21	0.011	0.001	23	0.63	0.010	0.002	0.011	0.001	0.03	0.85		0.88	
		Detergent	0.006	0.005	16	0.023	0.005	20	101	0.006	0.005	0.023	0.005	**5.85**	**0.02**	**0.15**	**0.046**	**↑**
	NHE9	Aqueous	0.017	0.002	25	0.012	0.001	25	0.21	0.016	0.002	0.013	0.002	1.92	0.17		0.13	
		Detergent	0.008	0.002	18	0.014	0.002	19	2.89	0.008	0.002	0.014	0.002	**4.22**	**0.048**	**0.11**	**0.04**	**↑**
	RACK1	Aqueous	0.055	0.006	25	0.051	0.004	24	0.45	0.054	0.005	0.051	0.005	0.18	0.67		0.69	
		Detergent	0.03	0.03	17	0.14	0.03	22	149	0.03	0.03	0.14	0.03	**7.2**	**0.01**	**0.16**	**0.04**	**↑**

Direction of change and effect size (η^2^_p_) are only provided for findings with *p*  < 0.1. Significant findings (*p* < 0.05) are bolded.

*GLM* general linear model, *COMP* comparison, *SZ* schizophrenia, *DOC* direction of change in schizophrenia relative to comparison group, *η*^*2*^_*p*_ partial Eta squared, *EMM* estimated marginal mean, *SEM* standard error of the mean, *GO* glycosylated oligomer, *HGM* highly glycosylated monomer, *CGM* core glycosylated monomer.

Bold values represent significant findings.

Given that tissue pH had the greatest number of associations with our dependent measures in our GLMs, a secondary Pearson correlational analysis was conducted comparing associations between tissue pH and our dependent measures within each group (COMP and SZ). To assess for potential antipsychotic effects on our dependent measures, we conducted another secondary analysis wherein we compared those individuals in the SZ group on antipsychotics at their time of death (*n* = 16) to those in the SZ group off of antipsychotics at their time of death for at least 6 weeks (*n* = 7) using GLMs controlling for the same covariates as the main analysis followed by bootstrapping. Measures from rat cortical tissue were analyzed by two-tailed unpaired *t-*tests. All analyses were conducted in SPSS, Version 29 (IBM) while GraphPad Prism, Version 9.3.1 (GraphPad Software, La Jolla, CA) was used for presentation of data. For all statistical tests, *α* = 0.05.

## Results

### DLPFC expression of NHEs in SZ and COMP

**Fig. 2:**
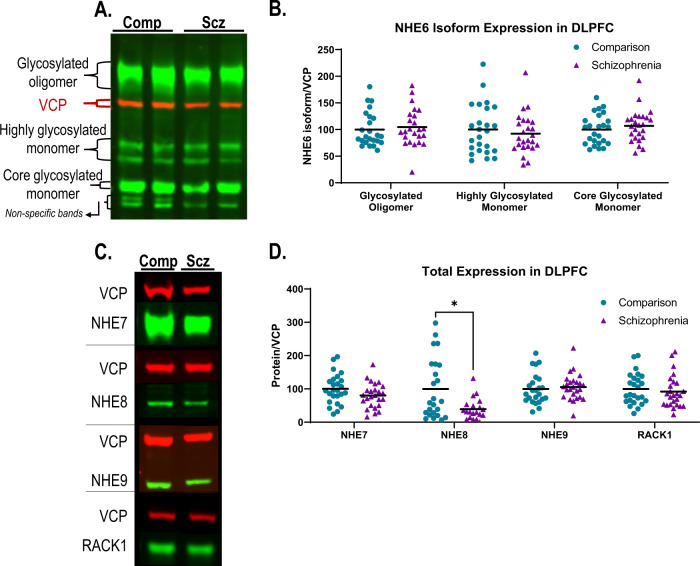
Protein expression of NHE6-NHE9 and RACK1 in dorsolateral prefrontal cortex (DLPFC) as measured by Western blot in schizophrenia (SZ) and comparison (COMP) subjects. **A** Representative Western blot of NHE6 immunoreactive bands demonstrating three main isoforms: glycosylated oligomers, highly glycosylated monomers, and core glycosylated monomers. **B** Quantification of each NHE6 isoform normalized to intralane valosin-containing protein (VCP) did not reveal any difference in expression between SZ and COMP. **C** Representative Western blots of NHE7-NHE9 and RACK1 in SZ and COMP DLPFC. **D** Quantification of NHE7-NHE9 and RACK1 protein expression normalized to intralane VCP demonstrates a significant decrease in NHE8 expression in SZ DLPFC and no change in NHE7, NHE9, or RACK1 expression between groups. Lines represent group means. *p < 0.05.

NHE6 expression in postmortem human cortex revealed multiple bands on Western blots (Fig. 2A). A blocking experiment utilizing recombinant NHE6 protein revealed that three of these bands were specific for NHE6 (Supplementary Fig. 1). This is consistent with prior characterization of NHE6 expression, and based on earlier reports likely represent glycosylated oligomers that do not dissociate under reducing conditions (~160 kD), highly glycosylated monomers (~60 kD), and core glycosylated monomers (~50 kD) [37, 70–73]. NHE7, NHE8, NHE9, and RACK1 expression in DLPFC all demonstrated a single band on Western blots at their predicted molecular weights (MW) of 75, 85, 73, and 35 kDa, respectively (Fig. 2C). There was significantly decreased expression of NHE8 (*p* = 0.02) in SZ DLPFC (Fig. 2D) while no change in the expression of all three forms of NHE6, NHE7, NHE9, and RACK1 was detected in SZ (Fig. 2B/D).

### RACK1 Co-IP of NHEs in SZ and COMP

**Fig. 3:**
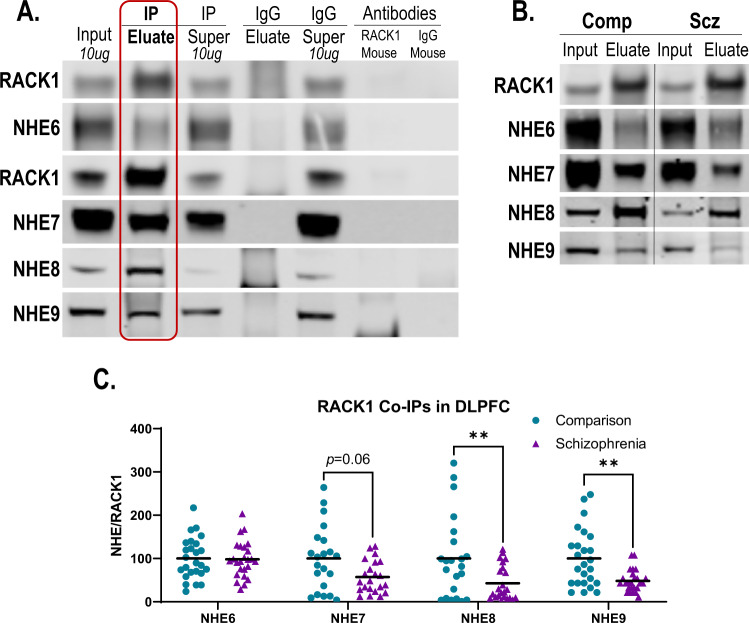
NHE association with RACK1 in schizophrenia dorsolateral prefrontal cortex (DLPFC). **A** Representative Western blots for RACK1 and NHE6 as well as RACK1 and NHE7-9 following co-immunoprecipitation (co-IP) with RACK1 antibody in human cortex. Co-IP of RACK1 enriches for RACK1 in IP eluate. NHE6-9 are all present in the RACK1 co-IP eluate suggesting interactions between these transporters and RACK1 in human cortex. No RACK1 enrichment was seen in a control co-IP with pre-immune, non-specific mouse IgG, demonstrating the specificity of co-IP with RACK1 antibody. In addition, RACK1 antibody and IgG alone did not produce immunoreactive bands at the molecular weights (MWs) of our assayed proteins. **B** Representative Western blots probing for RACK1 and NHE6-NHE9 in SZ and COMP DLPFC. **C** Quantification of NHE6-NHE9 normalized to intralane RACK1 in RACK1 IP eluate demonstrates decreased association between NHE8/NHE9 and RACK1 in SZ DLPFC with a nonsignificant decrease in NHE7 association with RACK1 between groups (*p* = 0.06). Lines represent group means. **p < 0.01. IP immunoprecipitation, Super supernatant, IgG immunoglobulin G, COMP comparison, SZ schizophrenia, co-IP co-immunoprecipitation.

In addition to changes in DLPFC NHE expression, increased interaction between these proteins and RACK1 can lead to their redistribution to the PM, which in the case of NHE6 significantly impacts endosomal pH [37, 38]. Thus, we measured interactions between RACK1 and each organellar NHE in SZ and COMP DLPFC by RACK1 co-IP. All of the organellar NHEs were present in eluate enriched for RACK1 (Fig. 3A). NHE8 and NHE9 were significantly decreased in SZ DLPFC RACK1 co-IP eluate (Fig. 3B/C) suggesting decreased interaction between RACK1 and these organellar NHEs in SZ DLPFC. No significant change in NHE6 or NHE7 in RACK1 co-IP eluate was identified in SZ (Fig. 3B/C).

### Aqueous- and detergent-soluble fraction expression of NHEs in SZ and COMP

**Fig. 4:**
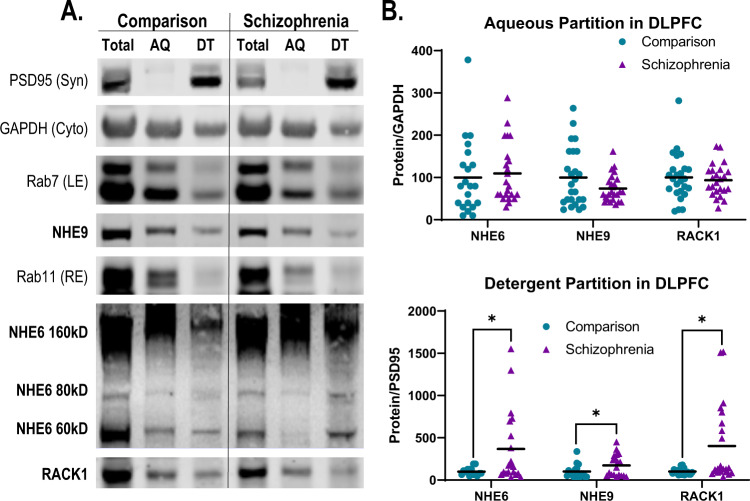
NHE Triton X-114 (TX-114) phase partitioning in schizophrenia dorsolateral prefrontal cortex (DLPFC). **A** Representative Western blots demonstrating TX-114 aqueous (AQ) and detergent (DT) phase partitioning of NHE6-NHE9, RACK1, recycling and late endosome markers Rab11 and Rab7, respectively, cytosolic marker GAPDH, and synaptic marker PSD95 in SZ and COMP DLPFC. PSD95 is enriched in the DT fraction while GAPDH, Rab7, and Rab11 are enriched in the AQ fraction. NHE6, NHE9, and RACK1 are present in both AQ and DT fractions but are all present in higher proportion in the AQ fraction. **B** Quantification of NHE6, NHE9, and RACK1 TX-114 AQ and DT partitioning in SZ and COMP. For the AQ fraction, NHE6, NHE9, and RACK1 were normalized to intralane GAPDH. NHE6, NHE9, and RACK1 were unchanged in the AQ fraction in SZ DLPFC. For the DT fraction, NHE6, NHE9, and RACK1 were normalized to intralane PSD95. NHE6, NHE9, and RACK1 were all increased in the DT fraction in SZ DLPFC. Lines represent group means. *p < 0.05. AQ aqueous phase, DT detergent phase, Syn synaptic marker, Cyto cytosolic marker, LE late endosome marker, RE recycling endosome marker.

We determined the intracellular distribution of organellar NHEs and RACK1 in SZ and COMP DLPFC by utilizing a TX-114 phase separation of tissue to yield a detergent-soluble (DT) fraction enriched for synaptic proteins (PSD95) and an aqueous-soluble (AQ) fraction enriched for cytosolic proteins (GAPDH) (Fig. 4A). As expected, markers for recycling (Rab11) and late (Rab7) endosomes, which are the intracellular targets of NHE6 and NHE9, respectively, were enriched in the AQ fraction, as were NHE6, NHE9, and RACK1 (Fig. 4A). While no significant change in NHE6, NHE9, or RACK1 was found in the AQ fraction (Fig. 4B), all were significantly increased in the SZ DT fraction (Fig. 4B). While we also attempted to measure NHE7 and NHE8 in AQ and DT fractions, NHE7 and NHE8 along with the Golgi markers STX6 and TGN38 were predominantly enriched in the DT fraction (Supplementary Fig. 2). This, unfortunately, precluded our ability to differentiate between NHE7 and NHE8 located at Golgi versus the synapse.

### Associations between tissue pH and dependent protein measures within groups

Perhaps not surprising given the physiological role of organellar NHEs in regulating intracellular and intracompartmental pH, our primary GLM analysis demonstrated the greatest number of associations between our dependent measures and tissue pH (Supplementary Table 3). To further explore this relationship, we conducted Pearson correlational analyses of pH associations with our dependent measures by group (COMP and SZ). Of note, associations between tissue pH and our dependent measures appear to have been driven in large part by strong associations within the COMP group (total DLPFC expression of core glycosylated monomer NHE6, NHE7, NHE8, and RACK1; RACK1 interaction with NHE8; and aqueous-soluble fraction expression of NHE6 and RACK1) that are not present in the SZ group (Supplementary Table 4, Supplementary Fig. 3). This suggests a possible loss of functional responsivity of organellar NHEs to tissue pH changes in SZ DLPFC.

### Effect of antipsychotic treatment on dependent measures

As those with schizophrenia are frequently on long-term antipsychotic therapy, we sought to determine whether being on antipsychotics at the time of death had an impact on any of our dependent measures by comparing those in the SZ group on antipsychotics at their time of death (*n* = 16) to those off of antipsychotics for at least 6 weeks at their time of death (*n* = 7). Of note, none of our dependent measures was found to be significantly different between those in the SZ group on antipsychotics versus those off of antipsychotics at their time of death (Supplementary Table 5).

### Effects of chronic haloperidol treatment in rats

Significant SZ-associated findings in this study were repeated in rats treated for 9 months with haloperidol to determine the effect of chronic antipsychotic treatment on these measures. There was no change detected in expression of NHE8 in haloperidol-treated rats compared to vehicle treatment (Supplementary Table 6; Supplementary Fig. 4A). There was also no change detected in the distribution of NHE6, NHE9, or RACK1 in cortical TX-114 AQ or DT fractions of rats treated with haloperidol (Supplementary Table 6; Supplementary Fig. 4B).

## Discussion

ER/Golgi-dependent protein PTM and trafficking are disrupted in SZ brain [3–12, 58]. The function of the secretory pathway organelles is greatly impacted by pH [13–15], and the organellar NHEs are major regulators of pH within these organelles [17, 18]. Despite evidence linking organellar NHEs with neurodevelopmental illnesses, female carriers of NHE6 loss of function mutations having an increased risk of primary psychotic disorders, and known disruptions in the secretory pathway in SZ, organellar NHEs have not been extensively characterized in SZ brain. In this study, we systematically examined organellar NHEs in SZ postmortem DLPFC, and identified multiple alterations which may have implications in SZ pathophysiology (Table 1).

We found a reduced expression of NHE8, which is targeted to mid/trans-Golgi stacks, in SZ DLPFC (Fig. 2C/D) suggesting that the Golgi may be especially vulnerable to pH perturbations in SZ DLPFC. Decreased expression of NHE8 is expected to favor increased Golgi acidity, which is associated with altered glycosylation and trafficking of proteins [14, 15]. NHE8, in addition to its localization to the Golgi, localizes in HeLa cells to multivesicular bodies, where it plays a key role in protein sorting and endosomal trafficking [74]. Furthermore, depletion of NHE8 leads to disrupted trafficking of early and late endosomes and increased protein degradation [74]. Thus, decreased expression of NHE8 in SZ DLPFC may suggest increased vulnerability of the cells in this region to altered trafficking and increased protein degradation.

In addition to changes in DLPFC expression of NHE8 in SZ, we also identified a trend toward decreased association of NHE7 with RACK1, which stabilizes organellar NHEs at the PM [37, 38], as well as a decreased association of NHE8 and NHE9 with RACK1 (Fig. 3B/C). While these findings may suggest decreased localization of NHE7, NHE8, and NHE9 at the PM, the intracellular localization of RACK1 must be considered before making this assumption. In addition to the PM, RACK1 is localized elsewhere including in the ribosome and centrosome [75–78]. Indeed, we found enrichment of RACK1 in the aqueous fraction of our TX-114 phase separation (Fig. 4A) suggesting increased cytosolic localization of RACK1 relative to PM localization in DLPFC. Thus, additional studies are needed to determine if RACK1 is complexing with NHEs elsewhere within the cell, and if this may be contributing to the overall decrease in NHE/RACK1 complexing we identified in SZ DLPFC.

To further assess the localization of organellar NHEs, we utilized a TX-114 phase partitioning approach, which enriches membrane proteins in the detergent phase and leaves cytosolic proteins in the aqueous phase (Fig. 4A). We found that in SZ DLPFC NHE6, NHE9, and RACK1 were significantly increased in the detergent phase enriched for the synaptic protein PSD95 (Fig. 4). These data suggest that NHE6 and NHE9 may be abnormally distributed from their resident endosomes to the PM in SZ DLPFC. In cell culture studies, redistribution of NHE6 from endosomes to the PM is associated with hyperacidification of endosomes and disruptions in their trafficking [37, 38]. Thus, a similar disruption of endosomal pH and trafficking may also occur in SZ DLPFC. Our finding of increased distribution of NHE6, NHE9, and RACK1 to the TX-114 DT phase may seem at odds with our finding that interactions between these proteins and RACK1 were either unchanged or decreased in the case of NHE6 and NHE9, respectively (Fig. 3B/C). However, as discussed above, the intracellular versus PM localization of RACK1 in DLPFC may need to be taken into account. In addition, a number of other binding partners are known to influence the PM expression of NHE6 and NHE9 independent of RACK1 [79–81], so interactions between organellar NHEs and these other binding partners will need to be assessed in future studies. It will also be important to systematically assess in greater detail the exact localization of organellar NHEs to fully understand the ways in which they are impacted in SZ brain.

### Caveats/limitations

Postmortem brain studies have inherent limitations. Our samples were from an older population, mostly male, and limited to the DLPFC region. As such, our findings may not generalize to other age groups or brain regions. Another limitation in SZ postmortem brain studies is accounting for potential confounding factors such as age, PMI, pH, and sex. To address this, we utilized a GLM approach to control for these factors (Supplementary Table 3), and as such, our major findings are unlikely to have been mediated by these confounding factors. It is worth noting that tissue pH was the factor most highly associated with our dependent measures (Supplementary Table 3) with this effect primarily driven by significant associations within the COMP group where tissue pH was significantly associated with seven of our dependent measures (Supplementary Table 4, Supplementary Fig. 3). This is in contrast to the SZ group where there were no significant associations between our dependent measures and tissue pH (Supplementary Table 4, Supplementary Fig. 3). This suggests that there may in fact be a loss of pH-responsivity of organellar NHEs in SZ DLPFC offering further evidence that mediators of intracellular and intracompartmental pH regulation are disrupted in schizophrenia brain.

An additional confounding factor in this study is the potential effect of antipsychotic treatment in the SZ group on our dependent measures. To address this, antipsychotic use in the SZ group prior to death was assessed and was not found to significantly impact any of our dependent measures (Supplementary Table 5). To further determine the effect of chronic antipsychotic exposure on protein changes independent of diagnosis, we assayed our dependent measures found to be significantly changed in SZ DLPFC in frontal cortex of rats chronically treated with haloperidol decanoate, and found that neither total expression of NHE8 nor TX-114 driven phase partitioning of NHE6, NHE9, or RACK1 were significantly different between haloperidol- and vehicle-treated groups (Supplementary Table 6, Supplementary Fig. 4), suggesting that our findings in SZ postmortem DLPFC were unlikely to have been mediated by chronic antipsychotic treatment alone.

Another limitation of the current study is that it was done in tissue homogenate, so we were not able to assess cell-type specific changes. Despite global changes in brain lactate and pH, cell types in the brain appear to be differentially affected in SZ with increasing evidence for disruption of the bioenergetic coupling between astrocytes and neurons [82]. There appears to be disruption of the astrocyte-neuron lactate shuttle, where lactate produced in astrocytes is transported to neurons, then converted back to pyruvate for entry into the tricarboxylic acid cycle. For instance, when mutant DISC1 is expressed only in astrocytes it is associated with decreased lactate, while iPSC-differentiated cortical neurons from individuals with SZ produce increased lactate [46]. This suggests that in SZ, astrocytes are unable to keep up with neuronal energy demands and that cortical neurons have an increased reliance on glycolysis for energy production. Thus, in future studies, it will be important to assess for cell-specific changes in organellar NHE expression, interactions, and localization.

### Summary/conclusion

We have characterized organellar NHEs in SZ DLPFC and identified multiple alterations, which may have implications for SZ pathophysiology (Table 1). We found decreased expression of NHE8, which could lead to altered endosomal trafficking and glycosylation. In addition, we identified altered localization of NHE6, NHE9, and RACK1 and decreased RACK1 interactions with NHE8 and NHE9 and a trend toward decreased RACK1 interactions with NHE7. Importantly, antipsychotic use prior to death did not appear to be associated with these dependent measures nor were these measures affected by chronic haloperidol administration in rats. The organellar NHEs are key pH regulators of secretory pathway organelles whose function is pH-dependent. Thus, the SZ-related changes in organellar NHEs we have identified are likely to be associated with aberrant function and activity of secretory pathway organelles and may offer a novel underlying mechanism for the altered PTM and trafficking seen in schizophrenia brain.

## Supplementary information

Supplementary Figure and Table Legends

Supplementary Figure 1

Supplementary Figure 2

Supplementary Figure 3

Supplementary Figure 4

Supplementary Table 1

Supplementary Table 2

Supplementary Table 3

Supplementary Table 4

Supplementary Table 5

Supplementary Table 6
