# Supplementary material for: Altered distribution and localization of organellar Na+/H+ exchangers in postmortem schizophrenia dorsolateral prefrontal cortex
Source: Transl Psychiatry. 2023 Feb 2;13:34. doi: 10.1038/s41398-023-02336-2 (PMC9895429; doi:10.1038/s41398-023-02336-2)
Supplement: Supplementary file 1 — Supplementary Figure and Table Legends [file 41398_2023_2336_MOESM1_ESM.docx]

**Supplementary Figure and Table Legends**

**Supplementary Figure 1.** Determination of NHE6 antibody specificity by a blocking experiment using recombinant NHE6 protein. (A) Western blot of NHE6 immunoreactivity under normal conditions (Supplementary Table 2). (B) Western blot of NHE6 immunoreactivity under normal conditions in the presence of 5x the amount of NHE6 recombinant protein (Abcam, ab161011) relative to NHE6 antibody. While there are several nonspecific bands coming in at <50 kD under blocking conditions, the signal for the remaining bands was completely blocked, suggesting that these bands are specific for the three main isoforms of NHE6: glycosylated oligomers, highly glycosylated monomers, and core glycosylated monomers. VCP was used as a loading control.

**Supplementary Figure 2.** NHE7, NHE8, and Golgi marker expression following Triton X-114 (TX-114) phase partitioning in human cortex. PSD95 is enriched in the detergent (DT) fraction, while GAPDH is enriched in the aqueous fraction. Golgi markers STX6 and TGN38 are enriched in the DT fraction. NHE7 and NHE8, which are targeted to trans-Golgi network and mid/trans-Golgi stacks, respectively, are also enriched in the DT fraction. Thus, it is not possible to determine the distribution of NHE7 and NHE8 between their Golgi targets and the synapse/plasma membrane using this method. Abbreviations: AQ, aqueous fraction; DT, detergent fraction; TGN, trans-Golgi network.

**Supplementary Figure 3.** Bivariate correlation plots of significant associations between tissue pH and dependent measures by group. Within the comparison group, there was a significant correlation between pH and (A) NHE6 core glycosylated monomer (CGM), (B) NHE7, (C) NHE8, (D) RACK1, (E) NHE8 in RACK1 co-immunoprecipitation (co-IP), (F) NHE6 in TX-114 aqueous fraction, and (G) RACK1 in TX-114 aqueous fraction. However, there were no significant correlations between pH and any of these measures within the schizophrenia group. This difference was particularly pronounced for (C) NHE8 and (E) NHE8 in RACK1 co-immunoprecipitation. Lines represent linear regressions of each dependent measure within each group. Abbreviations: CGM, core glycosylated monomer; co-IP, co-immunoprecipitation; VCP, valosin-containing protein.

**Supplementary Figure 4.** NHE expression in frontal cortex of rats treated chronically with haloperidol. (A) Quantification of NHE8 expression normalized to intralane VCP in frontal cortex of rats treated chronically with haloperidol or vehicle did not differ between groups. (B) Quantification of NHE6, NHE9, and RACK1 in aqueous (AQ) and detergent (DT) fractions following Triton X-114 partitioning in frontal cortex of rats treated chronically with haloperidol or vehicle. For the AQ fraction, NHE6, NHE9, and RACK1 were normalized to intralane GAPDH, while for the DT fraction, they were normalized to intralane PSD95. There were no differences in NHE6, NHE9, or RACK1 expression between groups in either the AQ or DT fractions. Lines represent group means and error bars represent the standard error of the mean (SEM). Abbreviations: AQ, aqueous; DT, detergent.

**Supplementary Table 1.** Subject demographics. Abbreviations: PMI, postmortem interval; M, male; F, female; Min, minimum; Max, maximum. None of the group differences were significant.

**Supplementary Table 2.** Antibodies/antisera and assay conditions. Abbreviations: LI-COR, LI-COR Odyssey Blocking Buffer; TBST, Tris-buffered saline + 0.05% Tween-20; NFDM, non-fat dry milk; BSA, bovine serum albumin; ON, overnight; RT, room temperature; hr, hour.

**Supplementary Table 3.** Fixed factors (sex), covariates (age, tissue pH), and interaction terms included in univariate general linear models (GLMs). Abbreviations: GO, glycosylated oligomer; HGM, highly glycosylated monomer; CGM, core glycosylated monomer.

**Supplementary Table 4.** Pearson correlational analyses of associations between tissue pH and dependent measures by group. Abbreviations: GO, glycosylated oligomer; HGM, highly glycosylated monomer; CGM, core glycosylated monomer.

**Supplementary Table 5.** Comparison of dependent measures in schizophrenia group on antipsychotics at time of death to those off antipsychotics for at least 6 weeks at time of death. Abbreviations: EMM, estimated marginal mean; SEM, standard error of the mean; GO, glycosylated oligomer; HGM, highly glycosylated monomer; CGM, core glycosylated monomer.

**Supplementary Table 6.** Summary of findings in chronically antipsychotic-treated rats. Group values are reported as means ± SEM. None of the group differences were significant.
