# Supplementary material for: Altered distribution and localization of organellar Na+/H+ exchangers in postmortem schizophrenia dorsolateral prefrontal cortex
Source: Transl Psychiatry. 2023 Feb 2;13:34. doi: 10.1038/s41398-023-02336-2 (PMC9895429; doi:10.1038/s41398-023-02336-2)
Supplement: Supplementary file 2 — Supplementary Figure 1 [file 41398_2023_2336_MOESM2_ESM.pdf]

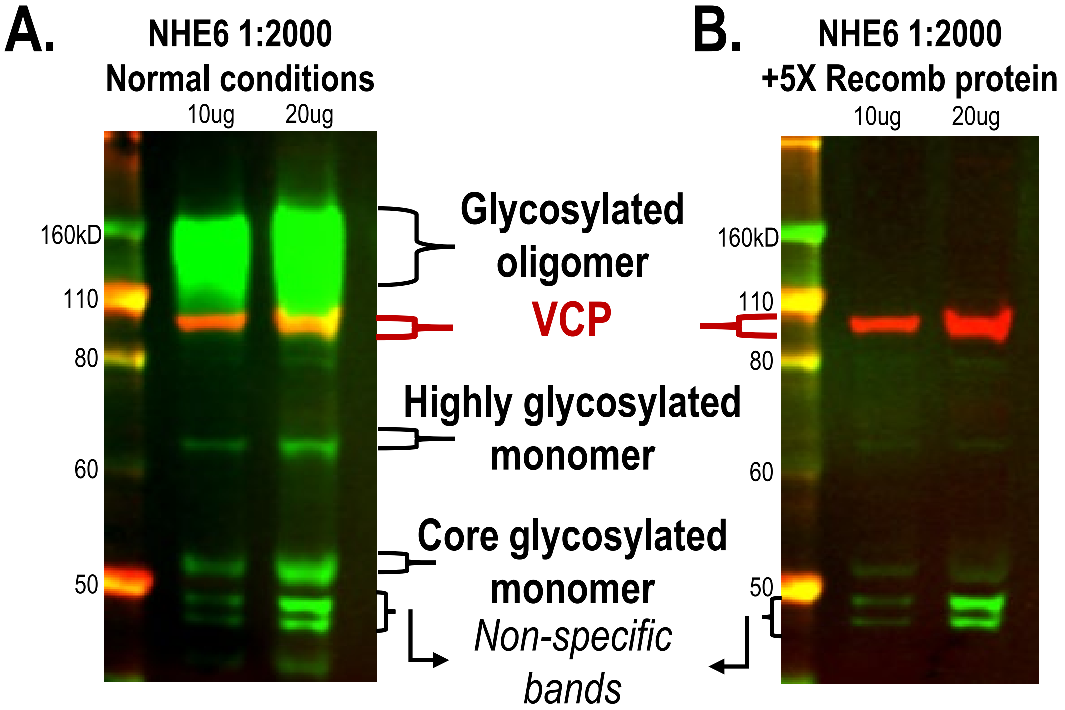
A.
NHE6 1:2000
Normal conditions
10ug
20ug
160kD
110
80
60
50
B.
NHE6 1:2000
+5X Recomb protein
10ug
20ug
160kD
110
80
60
50
Glycosylated oligomer
VCP
Highly glycosylated monomer
Core glycosylated monomer
Non-specific bands
