# Supplementary figures and images for: Altered distribution and localization of organellar Na+/H+ exchangers in postmortem schizophrenia dorsolateral prefrontal cortex
Source: Transl Psychiatry. 2023 Feb 2;13:34. doi: 10.1038/s41398-023-02336-2 (PMC9895429; doi:10.1038/s41398-023-02336-2)

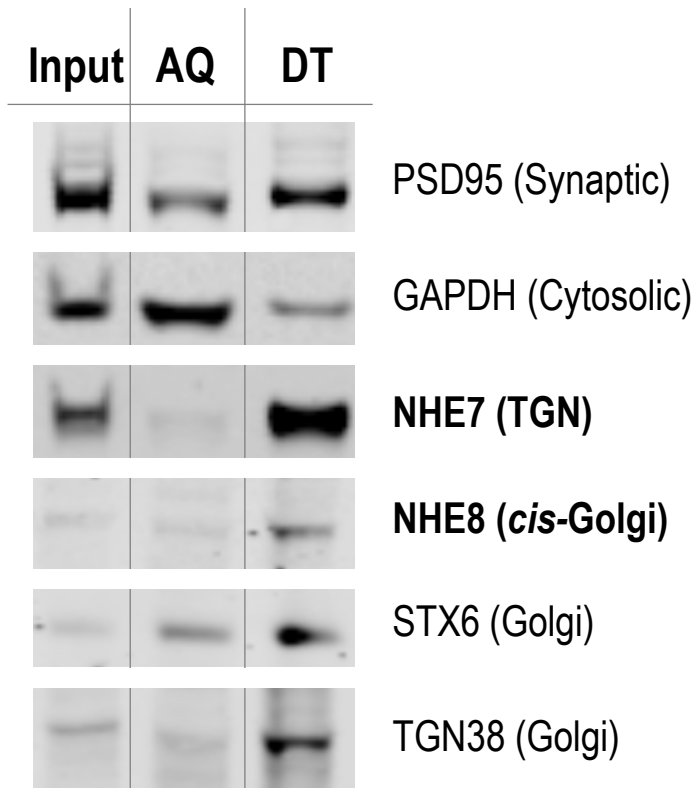

Supplement: Supplementary file 3 — Supplementary Figure 2 [file 41398_2023_2336_MOESM3_ESM.pdf]

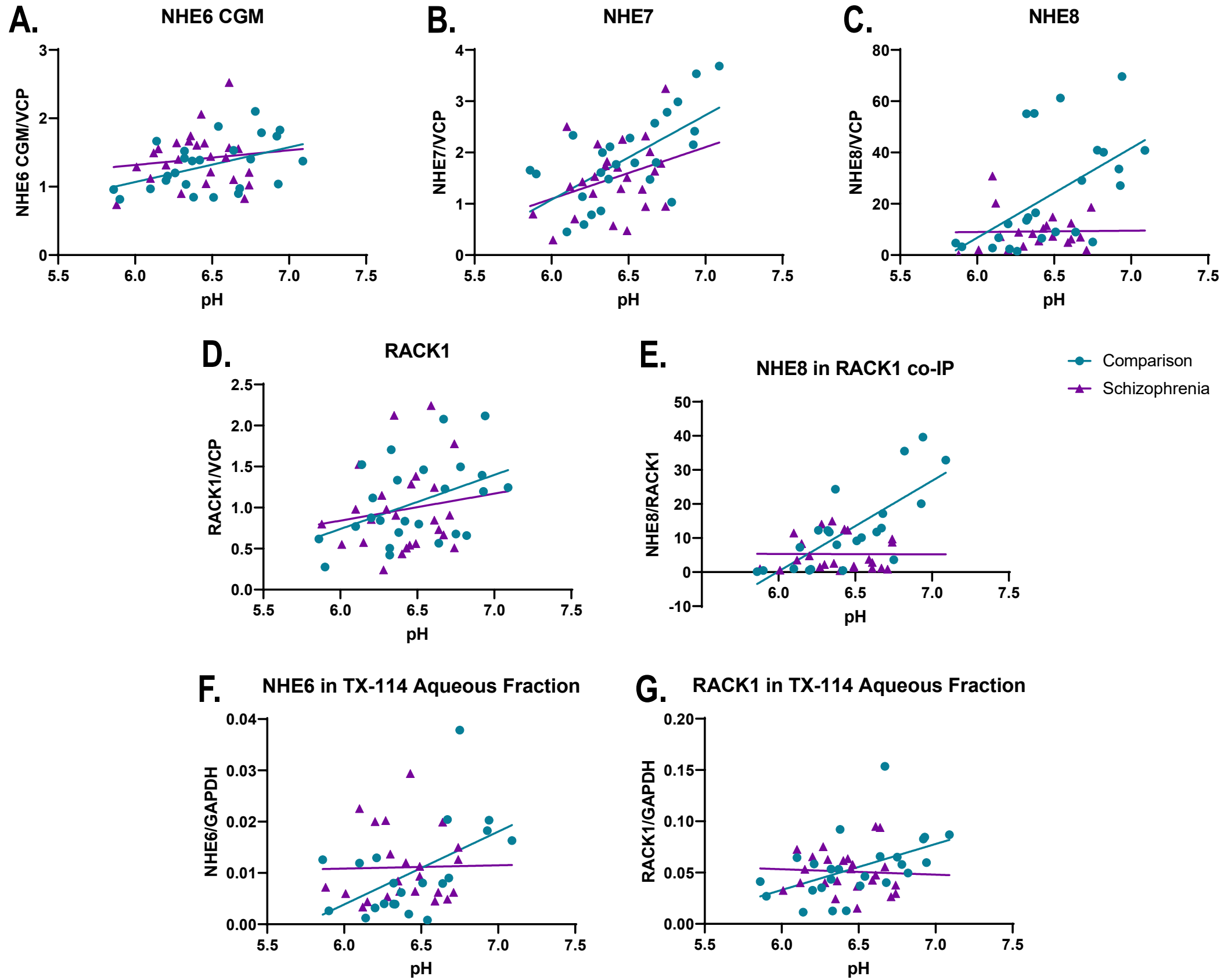

Supplement: Supplementary file 4 — Supplementary Figure 3 [file 41398_2023_2336_MOESM4_ESM.pdf]
