# Supplementary material for: Altered distribution and localization of organellar Na+/H+ exchangers in postmortem schizophrenia dorsolateral prefrontal cortex
Source: Transl Psychiatry. 2023 Feb 2;13:34. doi: 10.1038/s41398-023-02336-2 (PMC9895429; doi:10.1038/s41398-023-02336-2)
Supplement: Supplementary file 5 — Supplementary Figure 4 [file 41398_2023_2336_MOESM5_ESM.pdf]

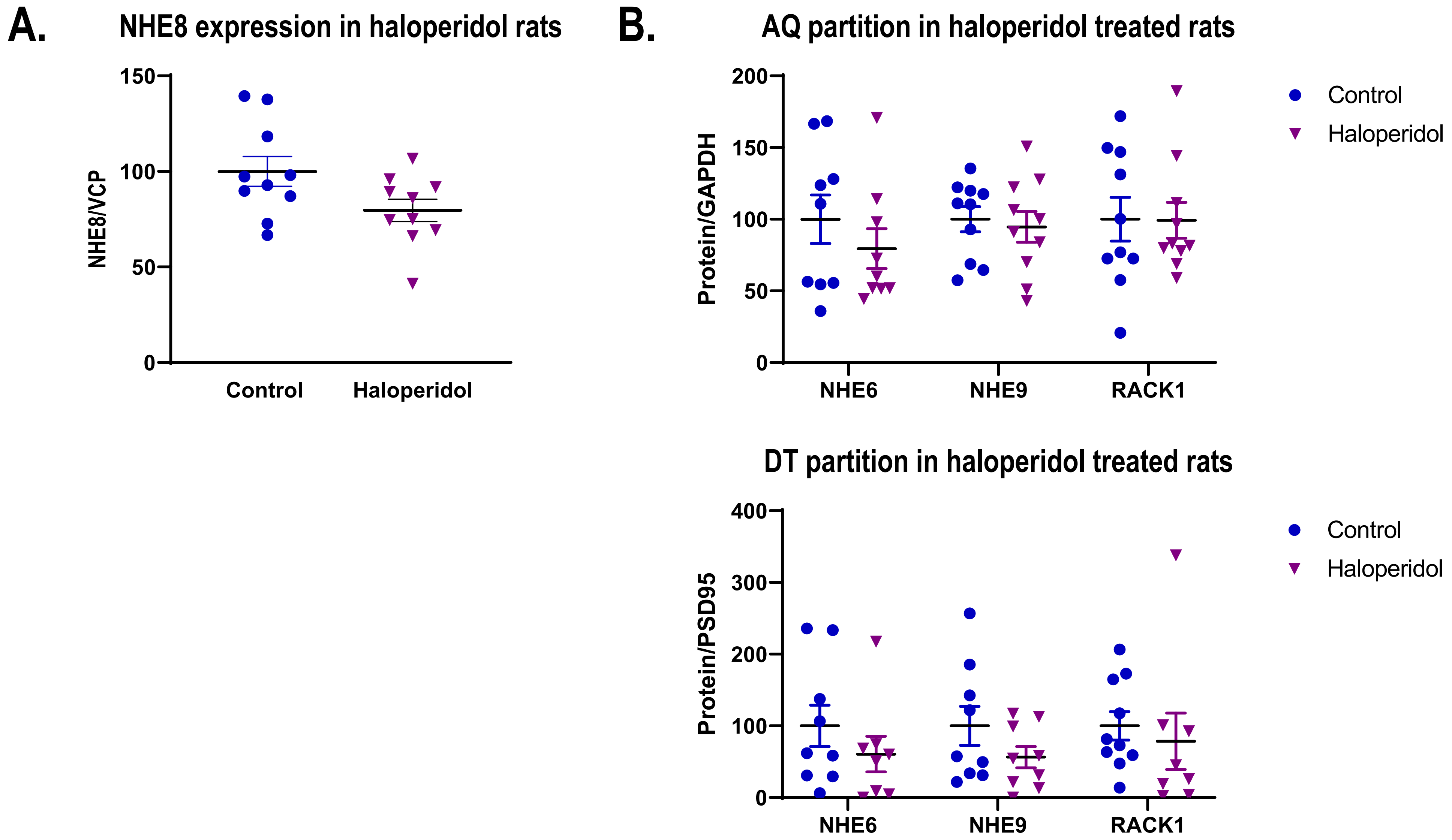
A.
NHE8 expression in haloperidol rats
NHE8/VCP
150
100
50
0
Control
Haloperidol
B.
AQ partition in haloperidol treated rats
Protein/GAPDH
200
150
100
50
0
NHE6
NHE9
RACK1
Control
Haloperidol
DT partition in haloperidol treated rats
Protein/PSD95
400
300
200
100
0
NHE6
NHE9
RACK1
Control
Haloperidol
